# Morphometric assessment of pterosaur jaw disparity

**DOI:** 10.1098/rsos.172130

**Published:** 2018-04-25

**Authors:** Charlie A. Navarro, Elizabeth Martin-Silverstone, Thomas L. Stubbs

**Affiliations:** 1 School of Earth Sciences, University of Bristol, Wills Memorial Building, Queen's Road, Bristol BS8 1RJ, UK; 2 Ocean and Earth Science, National Oceanography Centre, University of Southampton, European Way, Southampton SO14 3ZH, UK

**Keywords:** pterosauria, geometric morphometrics, disparity, morphospace

## Abstract

Pterosaurs were a successful group of Mesozoic flying reptiles. They were the first vertebrate group to achieve powered flight and varied enormously in morphology and ecology, occupying a variety of niches and developing specialized feeding strategies. Ecomorphological principles suggest this variation should be reflected by great morphological diversity in the lower jaw, given that the mandible served as the primary apparatus for prey acquisition. Here we present the first study of mandibular shape disparity in pterosaurs and aim to characterize major aspects of variation. We use a combination of geometric morphometric approaches, incorporating both outline analysis using elliptical Fourier analysis and semi-landmark approaches. Our results show that morphological convergence is prevalent and many pterosaurs, belonging to diverse dietary groups and subclades, overlap in morphospace and possessed relatively simple ‘rod-shaped’ jaws. There is no clear trend of size distributions in pterosaur mandibular morphospace, and larger forms are widely distributed. Additionally, there is limited functional signal within pterosaur lower jaw morphospace. Instead, the development of a large anterior ventral crest represents the major component of disparity. This suggests that a socio-sexual trait was a key driver for innovation in pterosaur lower jaw shape.

## 1. Introduction

**Figure 1.**
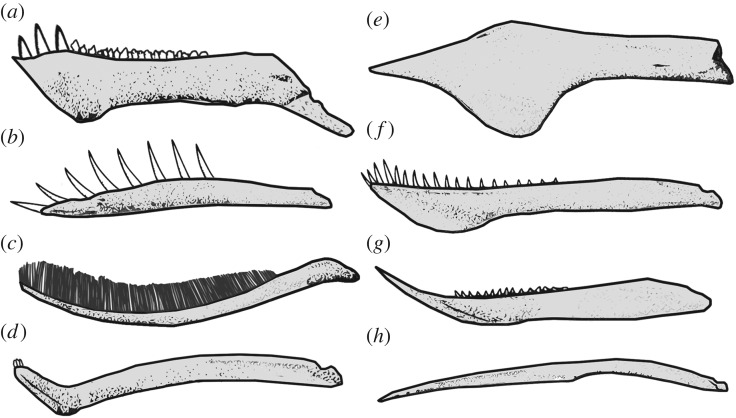
Morphological variation in pterosaur lower jaws. The illustrated taxa are (*a*) *Raeticodactylus*, (*b*) *Rhamphorhynchus*, (*c*) *Pterodaustro*, (*d*) *Cycnorhamphus*, (*e*) *Tapejara*, (*f*) *Anhanguera*, (*g*) *Dsungaripterus* and (*h*) *Quetzalcoatlus*. The selected jaws are illustrated to highlight shape disparity in the lateral profile and are not plotted to scale. Jaws (*b*), (*c*) and (*e–h*) are based on illustrations by Jaime Headden.

Pterosaurs were the first vertebrate group to achieve powered flight and include the largest animals to ever fly, making them an extremely important fossil group to understand [1,2]. During their long evolutionary history, ranging from the Late Triassic (*ca* 210 Ma) to the end of the Cretaceous (66 Ma), pterosaurs filled many ecological niches [3,4]. These included arboreal [5], coastal [6] and terrestrial forms [7,8], with a great variety of diets, such as insectivory, piscivory, carnivory, hypothesized frugivory and even feeding on small marine organisms through filter feeding [8–11]. This ecological variation is reflected by incredible morphological diversity, including many anatomical specializations in feeding-related morphology of the skull, mandible and dentition [12] (figure 1).

Mandibular morphology is expected to have a strong dietary and ecological signal, with feeding mode predicted to be a strong determinant for variation [13–18]. In pterosaurs, the mandible served as the primary apparatus for food acquisition and processing, whereas the skull also accommodated the brain, sensory systems and, in some cases, extremely extravagant cranial crests [19]. Recently, Zhou *et al*. [20] investigated ecomorphological innovations during pterosaur evolutionary history. They suggest that early pterosaurs were either insectivores or piscivores, while other feeding habits arose during the Cretaceous. Zhou *et al*. [20] also suggest that different feeding habits can be distinguished in empirical morphospaces derived from discrete descriptive characters. Some dsungaripterids had very distinct lower jaws with durophagous posterior dentition, and the lower jaw of the genus *Dsungaripterus* had a distinctly upturned anterior tip. Ornithocheirids and anhanguerids had forward-facing teeth anteriorly and long, slender lower jaws, probably aiding the capture of fish [21]. Large-bodied derived azhdarchids, such as *Quetzalcoatlus*, had slender lower jaws and may have been carnivorous, preying on small vertebrates such as juvenile dinosaurs [8]. *Pterodaustro* exhibited a geometrically bizarre mandibular morphology with a curving ‘scoop-shape’, densely packed with needle-like teeth, proposedly used for filter feeding [22]. The mandible of *Cycnorhamphus* was also unconventional, with pronounced curvature in the anterior symphyseal region and peg-like teeth restricted to the tip [23] (figure 1).

Pterosaur jaw shape disparity has never been considered in a macroevolutionary context. It is not known whether morphological divergence is the overriding trend, or if convergence is prevalent, particularly for taxa with similar dental morphologies, or sizes—owing to aerodynamic constraints or possibly phylogenetic signal.

The relationship between aerodynamics, pterosaur anatomy and skeletal specialization has been the subject of much research [24–27], with emphasis placed on the importance of large body size and an aerial lifestyle [28–30]. The diversification of pterosaurs gave rise to an exceptional range of body sizes, with wingspans ranging from 1 to 10 m [31]. Size is often an important component for determining variation in other traits, and the relationship between size and shape (allometry) is well documented [32–34]. Mandible size may have been an important factor for shape innovation in pterosaurs, potentially acting as both a constraint, on less aerodynamically efficient forms, and/or a catalyst for variation, by expanding the range of prey sizes that could be consumed.

Morphological diversity (disparity) can be quantified and visualized in several ways, including geometric morphometric landmarks and outlines (e.g. [34–36]), functionally relevant measurements and ratios [37,38] and discrete descriptive characters [20,39,40]. Previous pterosaur disparity studies have been conducted, which assessed disparity of the skull with geometric landmarks [41], skeletal disparity based on discrete characters [2] and ecomorphological variation using continuous and discrete cranial characters [20], but mandible shape has never been considered. Mandibular disparity has been the focus of studies exploring ecomorphological and functional variation in a macroevolutionary context in other groups, including arthrodires [13,42], early tetrapods [43], mammals [17], crurotarsans [35] and dinosaurs [18].

Here we examine mandibular disparity in pterosaurs using two morphometric approaches: outline analysis based on elliptical Fourier analysis (EFA) and semi-landmark (SLM) analysis. Our aim is to explore shape variation and produce empirical morphospaces, characterizing major aspects of morphological variation in the pterosaur lower jaw. We examine the distribution of pterosaur subgroups in morphospace to test the prevalence of morphological divergence and convergence, and investigate potential constraints, such as jaw size and feeding guild (guided by dental morphology). We test whether morphological disparity in the lower jaw is evenly distributed throughout the clade, or whether particular dietary guilds and subclades show higher disparity. Using two complementary methodological techniques, we also provide a comparative assessment of whether different morphometric protocols converge on a common signal.

## 2. Methods

### 2.1. Taxon sampling

A sample of 46 pterosaur lower jaws was used to investigate morphological disparity in the clade (electronic supplementary material). Complete laterally preserved mandibles are relatively rare in the pterosaur fossil record, but there is a sufficient enough number to sample representatives of all major subclades, time intervals and ecologies, except for the enigmatic anurognathids. Our sample size and scope are comparable with previous disparity studies (e.g. [41]). Individual specimens were used to represent each taxon, and lateral jaw images were sourced during collection visits and from figures and reconstructions in the literature (see electronic supplementary material). For comparative purposes, the specimens were grouped into taxonomic subgroups and dental guilds (electronic supplementary material). Taxonomic group selection and membership was guided by the large-scale phylogenetic analyses of Andres & Myers [44] and Andres *et al*. [45]. Dental guild selection and membership were decided through comparative assessment, based on eight guilds (see electronic supplementary material). These comparisons were used to investigate if similar dental guilds were associated with lower jaw shape, with the null expectation that taxa with similar teeth would have similar feeding habits, and therefore also similar lower jaw shapes.

### 2.2. Morphometric analyses

**Figure 2.**
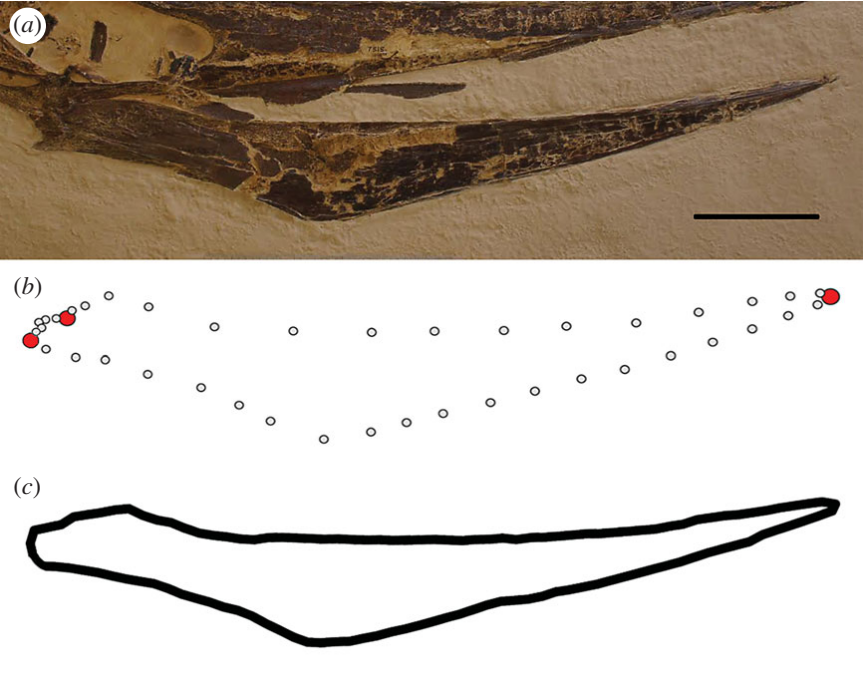
Landmark and outline protocols for the geometric morphometric analyses. (*a*) Mandible of *Pteranodon* AMNH FARB 7515 in lateral view. (*b*) Schematic illustrating the positions of landmarks (red) and SLM curves (grey). (*c*) Schematic illustrating the shape represented by a closed outline. Scale bar, 10** **cm.

This study focuses on the quantification and visualization of overall geometric variation in the lateral profile of the pterosaur lower jaw (figures 1 and 2). In pterosaur jaws, due to both biological and taphonomic reasons, there are few clearly identifiable homologous points which would serve as robust anatomical loci in standard landmark-based geometric morphometrics [46]. We therefore use two methodological approaches specifically designed to capture geometric shape variation where there is a lack of fixed homologous points: two-dimensional outline analysis based on EFA and SLM analysis.

For the EFA, the 46 lower jaws were converted into digitized outlines forming closed curves (figure 2*c*). To remove the noise effects of size, position and rotation, the jaw outlines were manually centred, scaled and aligned along the *x*-axis. The outlines were then defined by a standard number of *x*- and *y*-coordinate points (500) and a consistent starting position for the first coordinate was designated (southwards of the centroid) [47]. In EFA, a set of harmonics (sine and cosine functions) are generated through Fourier decomposition, aiming to imitate the target shapes. Each harmonic yields four Fourier coefficients describing the shapes (sine and cosine amplitudes for both *x*- and *y*-projections). Here, 11 harmonics were retained for each jaw (44 Fourier coefficients), encapsulating 99% of the cumulative power. These coefficients were then subjected to principal components analysis (PCA) to ordinate the sampled jaws and explore major aspects of the geometric variation. The outline analyses were performed in the R package *Momocs* [47].

For the SLM analysis, we used a combined landmark/SLMs approach (figure 2*b*). Only three homologous landmarks could be consistently identified: (i) the most anterior point of the dorsal surface of the lower jaw, (ii) the point at which the lower jaw articulates with the cranium and (iii) the most posterior point of the lower jaw at the end of the retroarticular process (figure 2*b*). To capture the entire shape of the mandible, 40 SLMs were positioned on three curves: 15 points between landmarks 1 and 2 (dorsal surface of the mandible), five points between landmarks 2 and 3, and 20 points between landmarks 3 and 1 (ventral surface of the mandible). The fixed and SLMs were applied digitally to all specimens using tpsDig [48]. To remove the noise effects of size, position and rotation, all 43 landmarks were aligned using a generalized Procrustes analysis (GPA) [46]. During the GPA, the SLMs were allowed to iteratively slide to minimize Procrustes distances between each specimen and the average shape [49]. The resulting set of aligned landmark coordinates were then subjected to PCA, to examine key components of shape variation. GPA and PCA were conducted in PAST [50] and the R package *geomorph* [51].

We assess similarity in how the morphometric techniques quantify shape variation using correlations of pairwise distances. Mantel tests were performed on Euclidean distance matrices derived from the 44 Fourier coefficients (EFA) and the Procrustes-aligned landmark coordinates (SLM). Strong correlation between the distance matrices would imply that both techniques capture similar morphological differences. Calculations were performed in the R package *ade4* [52] and *vegan* [53].

### 2.3. Morphospace occupation

Statistical tests were used to identify significant differences in morphospace occupation between the taxonomic groups and dental guilds. We use a series of non-parametric multivariate analysis of variance (NPMANOVA) tests. These permutation tests were performed on principal component (PC) scores from axes incorporating 95% of overall variation. Statistically significant differences, and therefore contrasting morphospace occupation, are denoted by *p* < 0.05 after Bonferroni's corrections for multiple comparisons. NPMANOVA tests were performed in PAST [50].

To explore phylogenetic branching patterns within the morphospaces, a pterosaur phylogeny was superimposed. We used the large-scale phylogeny from Andres *et al*. [45] because it is more recent than the alternative commonly used phylogenies of Unwin [54] and Kellner and co-workers [55], and it has been used in other macroevolutionary studies (e.g. [31]). This tree also maximized taxonomic coverage; however, the following taxa had to be added informally based on placements described in the literature: *Barbosania gracilirostris*, *Bergamodactylus wildi*, *Caiuajara dobruskii*, *Hamipterus tianshanensis*, *Ikrandraco avatar* and *Jianchangnathus robustus*. The ‘Painten pro-pterodactyloid’ of Tischlinger & Frey [56] was excluded from the phylomorphospaces because its phylogenetic position is uncertain. The tree was time-calibrated with the midpoint of each taxon's stratigraphic range, using the equal dating method [57,58]. Absolute ages for the occurrences are from Benson *et al*. [31] and Gradstein *et al*. [59]. The positions of internal nodes in the phylomorphospaces were estimated using maximum-likelihood approaches in the R package *phytools* [60].

### 2.4. Size and shape

Statistical tests for allometry were based on multivariate regressions of the jaw shape variables and centroid size [32]. For the outline-based shape data, multivariate regression was performed on the 44 Fourier coefficients and log-transformed centroid size. For the landmark-based approach, allometry was assessed based on a multivariate regression of the Procrustes-aligned coordinates and log-transformed centroid size. Centroid size was calculated from the landmark coordinates using tpsRelw [61]. The statistical significance for each correlation test was assessed with permutation tests using 9999 iterations. All correlation tests were performed in the R package *geomorph* [51].

### 2.5. Disparity analyses

Comparative morphological disparity analyses were performed to test if taxonomic and dental subgroups showed equal morphological variation. Disparity was quantified based on the positions of taxa in the multivariate morphospaces. Taxa were binned into the taxonomic and dental groupings (electronic supplementary material) and PC scores from the axes that represented 95% of overall variation were used to quantify morphological variation. Disparity is here based on the sum of variances metric. Confidence intervals (95%), generated by bootstrapping with 10 000 replications, were used to test for statistically significant differences in disparity. All analyses were performed with the MDA Matlab® package [62] and in R [63].

## 3. Results

### 3.1. Pterosaur morphospace trends

**Figure 3.**
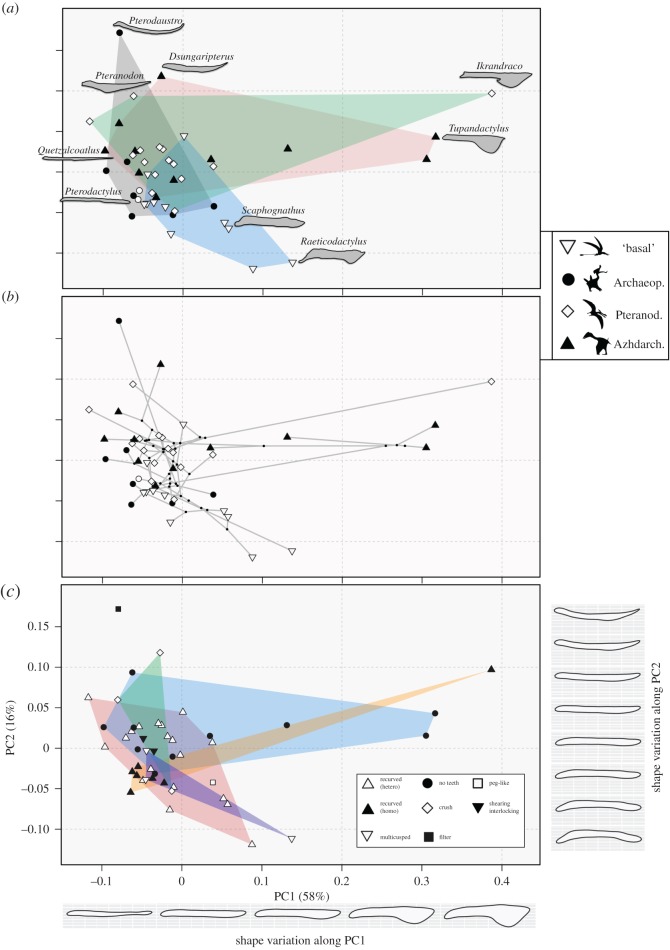
Pterosaur lower jaw morphospace based on elliptical Fourier outline analysis. Two-dimensional morphospace plots are based on the first two principal components axes. (*a*) Distribution of broad taxonomic groups (convex hulls) with numerous mandible shapes illustrated and labelled. (*b*) Phylomorphospace illustrating branching patterns. (*c*) Dental guild distributions (convex hulls). Taxonomic groups in (*a*) and (*b*) are ‘basal’ non-monofenestratan, Archaeopterodactyloidea, Pteranodontia and Azhdarchoidea. Shape variation along PC1 and PC2 is plotted. Silhouettes are taken from the following sources: ‘basal non-monofenestratan’ from http://phylopic.org by Dmitry Bogdanov, Archaeopterodactyloidea from http://phylopic.org/ by Matthew Martyniuk, Pteranodontia from http://phylopic.org/ by FunkMonk and Azhdarchoidea from Naish *et al*. [64].

**Figure 4.**
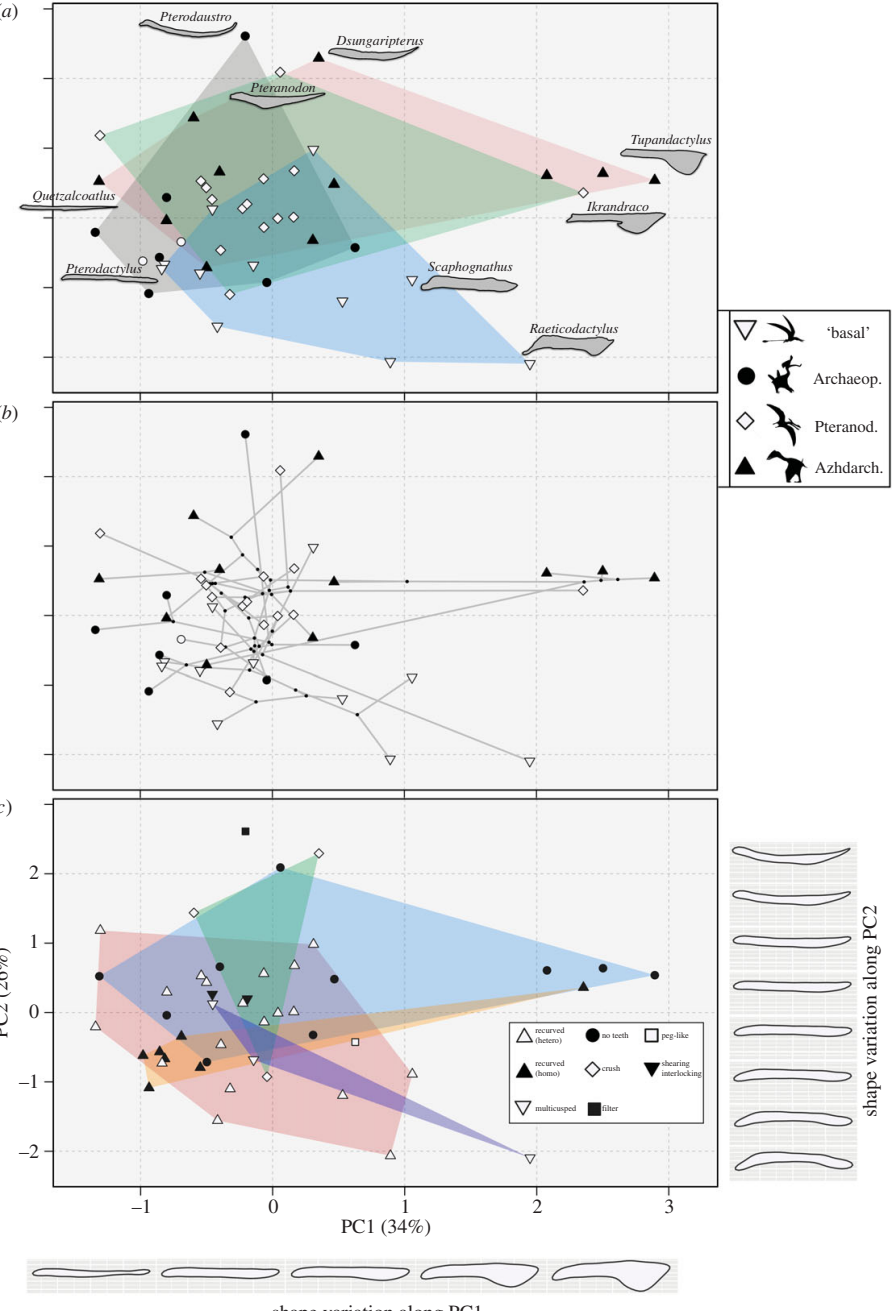
Pterosaur lower jaw morphospace based on landmark-based morphometric analysis. Two-dimensional morphospace plots are based on the first two principal components axes. (*a*) Distribution of broad taxonomic groups (convex hulls) with numerous mandible shapes illustrated and labelled. (*b*) Phylomorphospace illustrating branching patterns. (*c*) Dental guild distributions (convex hulls). Taxonomic groups in (*a*) and (*b*) are ‘basal’ non-monofenestratan, Archaeopterodactyloidea, Pteranodontia and Azhdarchoidea. Shape variation along PC1 and PC2 is plotted. Silhouettes are taken from the following sources: ‘basal non-monofenestratan’ from http://phylopic.org by Dmitry Bogdanov, Archaeopterodactyloidea from http://phylopic.org/ by Matthew Martyniuk, Pteranodontia from http://phylopic.org/ by FunkMonk, and Azhdarchoidea from Naish *et al.* [64].

In both outline and SLM-based analyses, most shape variation in pterosaur lower jaws can be summarized by the first two PC axes (figures 3 and 4). PC1 accounts for 58% of overall variance in the outline-based morphospace, and 34% in the landmark-based morphospace. The major element of shape change along PC1, in both spaces, is the development of a distinct and increasingly large ventral crest on the anterior part of the lower jaw, in addition to the overall changes in dorsoventral depth (figures 3*c* and 4*c*). Negative PC1 scores represent pterosaurs with slender lower jaws and no exaggerated structures (e.g. *Aetodactylus* and *Quetzalcoatlus*) (figures 3*a* and 4*a*), while taxa with the greater positive scores on PC1 have more robust jaws with massive ventral crests (e.g. *Tupandactylus* and *Ikrandraco*) (figures 3*a* and 4*a*). PC2 accounts for a further 16% of overall variance in the outline-based morphospace, and 26% in the landmark-based morphospace. PC2 represents the curvature of the mandible in both spaces (figures 3*c* and 4*c*). Mandibles with high positive PC2 scores are highly curved, with the anterior and/or posterior parts dorsally upturned relative to the jaw midpoint, resulting in most of the tooth row falling below the jaw–quadrate articulation. This morphotype is best exemplified by *Pterodaustro* and *Dsungaripterus* (figures 3*a* and 4*a*). Neutral PC2 scores represent taxa with straight lower jaws, whereas taxa with increasingly negative PC2 scores have jaws where the anterior and/or posterior parts are ventrally downturned relative to the midpoint, often resulting in a tooth row positioned above the jaw–quadrate articulation (e.g. *Raeticodactylus*).

Both analytical approaches recover the same major elements of shape change. Mantel tests confirm that the outline and landmark-based analyses quantify very similar morphological information. Comparison of pairwise Euclidean distance matrices derived from both shape datasets reveals a very strong and significant positive correlation (*z* = 0.9005; *p* = 0.001). Overall, the outline-based analyses capture morphological variation more succinctly than the landmark-based approach. The first two axes in the outline-based morphospace encapsulate 74% of overall variance, four PC axes are required to reach 90% of overall variance, with six axes needed to reach 95% and 12 axes to reach 99%. In comparison, the PC1–PC2 morphospace in the landmark-based approach encapsulates 60% of overall variance, while nine axes are required to reach 90%, 12 to reach 95% and 22 to incorporate 99%. The outline-based approach places greater emphasis on variation along PC1, while the landmark-based approach places greater weight on PC2. This is reflected by almost identical patterns of morphospace occupation and distribution, but variation in the spacing between taxa (figures 3 and 4).

One of the most striking trends in morphospace occupation is the large-scale overlap of many taxa from different taxonomic groupings in central morphospace (figures 3*a* and 4*a*). There is little evidence of discrete group clustering in both spaces. All major groups have divergent representatives that spread out from the central regions to more fully explore morphospace. The Archaeopterodactyloidea are found centrally, with primarily negative PC1 scores and neutral PC2 scores, representing straight and slender jaws. The notable exception is the outlying extreme *Pterodaustro*. Most pteranodontians are, again, positioned centrally (mainly Anhangueria), however *Ikrandraco* represents a distinct outlier, plotting close to the tapejarids at the extreme positive end of PC1 with a prominent crest. *Pteranodon* also falls separately from other, more derived, pteranodontians, and is found at a higher positive position along PC2. The main difference in Azhdarchoidea is between the neoazhdarchians and the tapejarids. Some neoazhdarchians, found in negative PC1 regions, such as *Quetzalcoatlus*, have slender and straight jaws, whereas the most extreme azhdarchoid morphology is shown by the tapejarids at the extreme positive end of PC1, with massively developed ventral crests. The phylomorphospaces further illustrate high levels of overlap and the clustering of internal nodes and branches (figures 3*b* and 4*b*). Only two divergent branching events can be identified. One major divergence is seen in derived tapejarids (Azhdarchoidea) into regions of high positive PC1 scores. The second major excursion is seen in a basal assemblage of non-pterodactyloids into negative PC2 space. Long branches leading to *Pterodaustro*, *Ikrandraco* and *Raeticodactylus* reveal how divergent they are compared with their parent clades and sister taxa (figures 3*b* and 4*b*).

Statistical tests for significant group separations reveal mixed results. Overall, pairwise comparisons among groups corroborate the visual inspections of morphospace and do not support discrete separation. Based on the outline analyses, no pairwise comparisons return statistically significant results (Bonferroni-corrected *p*-values range from 0.125 to 1). The same result is returned if narrower taxonomic divisions are used (Bonferroni-corrected *p*-values range from 0.051 to 1). Tests for separation in the landmark-based morphospace do return some statistically significant results, when comparing the ‘basal non-monofenestratan’ grouping to the pteranodontians (corrected *p* = 0.039) and Azhdarchoidea (corrected *p* < 0.001), and when comparing the pteranodontians to Azhdarchoidea (corrected *p* = 0.020). The only significant results when using narrower taxonomic subdivisions in the landmark data are found between the tapejarids and Anhangueria (corrected *p* = 0.011), and Anhangueria and Eudimorphodontidae (corrected *p* = 0.044).

There is no clear evidence for a strong link between jaw shape and dental morphology. Representatives from all dental guilds converge in central morphospace (figures 3*c* and 4*c*). Pterosaurs with recurved conical dentition and pronounced size heterodonty are the most common in our study, and such taxa are mostly positioned centrally, but they do show moderate expansion on both the major axes of variation. Taxa with homodont recurved conical dentition are clustered tightly, with the notable exception of *Ikrandraco* which is located close to edentulous tapejarids. Edentulous taxa are unexpectedly disparate and have wide morphospace occupation. No single jaw morphotype is characteristic of toothless taxa, and they occupy the extreme positions of positive and negative PC1 (*Quetzalcoatlus* and *Tupandactylus*) and positive PC2 (*Pteranodon*). Pterosaurs which possessed rarer and more specialized dental morphologies, including crushing, shearing, peg-like and multicusped, do not form separate clusters in mandible shape morphospace, but are instead all overlapping and close to taxa with simple recurved cones. *Pterodaustro*, the only taxon we sample with specialized filter-feeding dentition, is found at the extreme positive of PC2. Statistical tests for differences in morphospace occupation by dental groups return no significant results for the outline data. For the landmark data, significant differences are found between multicusped taxa and both edentulous forms (corrected *p* = 0.040) and taxa with heterodont and recurved cones (corrected *p* = 0.017), and between edentulous forms and taxa with heterodont and recurved cones (corrected *p* = 0.010).

### 3.2. The relationship between size and shape

**Figure 5.**
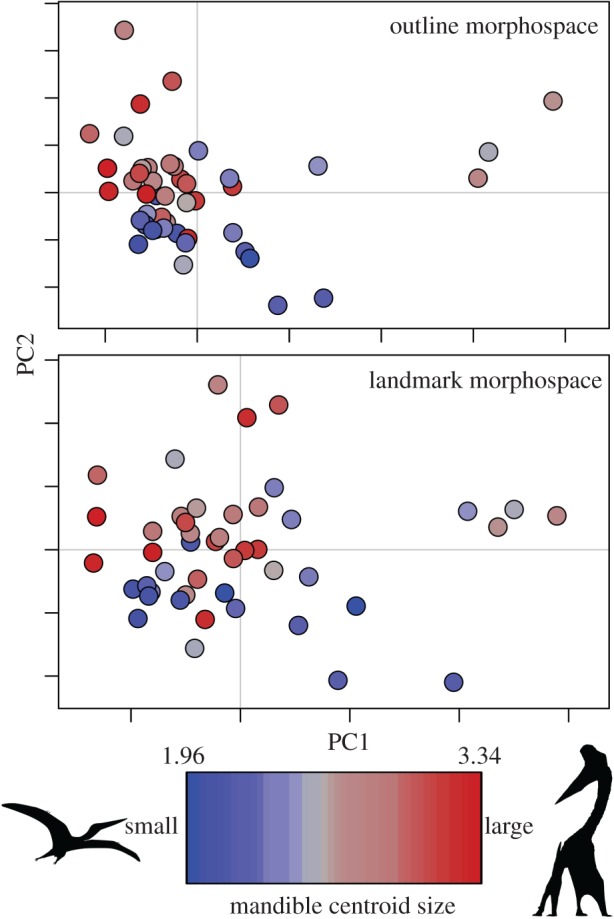
Distribution of lower jaw sizes within shape morphospaces. Pterosaur lower jaw morphospaces from the outline and landmark analyses are presented, with taxa plotted using a colour gradient according to log-transformed mandible centroid size. Left image is from http://phylopic.org by Gareth Monger, and the right image is by Mark Witton.

Lower jaw size has limited overall control on shape, and the position of taxa in morphospace (figure 5). Multivariate regression tests show there is a significant, but weak, relationship between mandible size and shape based on outline data (*p* = 0.041, *r*^2^ = 0.061) and SLMs (*p* = 0.002, *r*^2^ = 0.082). Size only accounts for 6% of shape variation in the outline data and 8% of variation in the landmark data after Procrustes superimposition. This trend is reinforced by visual inspections of distribution in morphospaces, where taxa of varying size are found distributed widely and frequently overlap (figure 5).

### 3.3. Disparity trends

**Figure 6.**
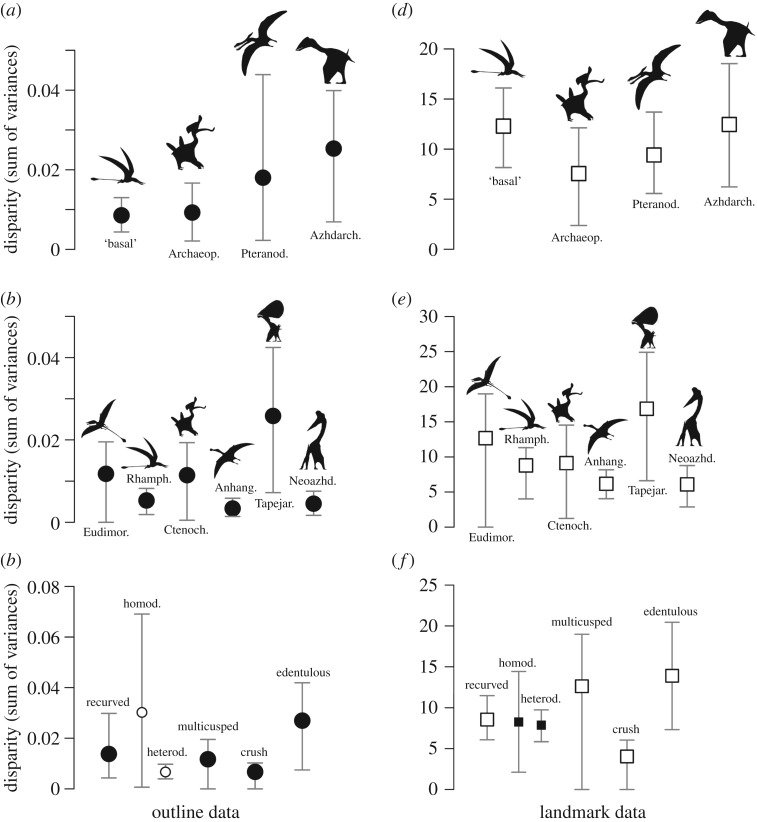
Mandibular morphological disparity in pterosaur subgroups, using outline data (*a*–*c*) and landmark data (*d*–*f*). Disparity is based on the sum of variances metric (circle and square symbols), with error bars representing 95% confidence intervals based on 1000 bootstrap replicates. Disparity is calculated for taxonomic groups (*a*,*b*,*d*,*e*) and dental guilds (*c*,*f*). The taxonomic groups used in (*a*) and (*d*) are ‘basal’ non-monofenestratan, Archaeopterodactyloidea, Pteranodontia and Azhdarchoidea. In (*b*) and (*e*) narrower subdivisions are used, including the groups Eudimorphodontidae, Ctenochasmatoidea, Rhamphorhynchidae, Anhangueridae, Tapejaridae and Neoazhdarchia. Disparity was calculated for the following dental guilds: recurved (all), recurved heterodont, recurved homodont, multicusped, ‘crushing’ and edentulous. Illustrative silhouettes are taken from the following sources: ‘basal non-monofenestratan’ from http://phylopic.org/ by Dmitry Bogdanov, Archaeopterodactyloidea from http://phylopic.org/ by Matthew Martyniuk, Pteranodontia from http://phylopic.org/ by FunkMonk, Azhdarchoidea from Naish *et al.* [64], Eudimorphodontidae by Corey Ford, Rhamphorhynchidae from http://phylopic.org/ by Dmitry Bogdanov, Ctenochasmatoidea from http://phylopic.org/ by Matthew Martyniuk, Anhangueridae by Nobu Tamura, Tapejaridae by Mark Witton, and Neoazhdarchia by Mark Witton.

Pterosaur subgroups have unequal mandibular shape disparity (figure 6). The Azhdarchoidea (incorporating tapejaromorphs and neoazhdarchians) have greatest disparity, based on both outline and landmark-based morphospace occupation. The Archaeopterodactyloidea consistently show low disparity. The ‘basal non-monofenestratan’ grouping shows high disparity in calculations based on the landmark-based morphospace (figure 6*d*), but not in the outline data (figure 6*a*). Confidence intervals associated with the disparity values consistently overlap. These large confidence intervals likely result from an abundance of outlying forms from each group and low sample sizes. Much of the disparity in Azhdarchoidea is contributed by the tapejarids, which are recovered as the most disparate group when narrower, less inclusive, subgroups are used (figure 6*b* and *e*). Most other small subgroups have indistinguishable disparity. Calculations from the landmark-based morphospace scores suggest the Eudimorphodontidae have relatively high disparity, however this is associated with a large confidence envelope resulting from a small sample size (*n* = 3).

Most dental guilds have similar mandibular shape disparity (figure 6*c* and *f*). Only edentulous forms consistently show higher disparity, based on both outline and landmark-based morphospace occupation. In the outline-based analyses, the homodont recurved dental group also has higher disparity (figure 6*c*), but this is associated with a large confidence envelope. This inflated disparity and confidence envelope results from the inclusion of the outlying taxon *Ikrandraco*. Similarly, in the landmark-morphospace calculations, the multicusped forms show elevated disparity (figure 6*f*), however this is once again associated with large confidence envelopes due to a small sample size (*n* = 3).

## 4. Discussion

Pterosaurs were ecologically diverse, but this study reveals that there is no clear relationship between lower jaw shape and other ecologically relevant characteristics, such as dental morphology and size (figures 3–5). Many pterosaurs, in phylogenetically distant subclades and with diverse diets, conformed to a mandible shape that was elongate, with a dorsoventrally thin dentary, short or absent retroarticular process, and the tooth row at the same level as the jaw joint. Convergence is the overriding trend, with a large degree of overlap in central morphospace, and no discrete clustering (figures 3 and 4). Divergent mandibular morphologies are represented by rare morphological extremes. These results contrast with Zhou *et al*. [20], who identified partitioning of feeding adaptations and clustering in pterosaur ecomorphospace. This discrepancy may, in part, be due to the large number of dental characters used by Zhou *et al*. [20]. Of the 34 morphological characters they sampled, 20 relate to dental variation. Therefore, it appears that a clearer ecomorphological signal is present in pterosaur dentition rather than jaw shape.

### 4.1. Mandibular crests

The development of a large anterior ventral crest represents the major element of mandibular shape disparity in pterosaurs (figures 3 and 4). Elaborate crests are known in several pterosaur lineages and are made up of bone or soft tissue, or a combination of both [65]. We sampled thirteen taxa with ventral crests, including five where the crest is greatly developed (*Tapejara*, *Tupandactylus*, *Ikrandraco*, *Caiuajara* and *Raeticodactylus*). Most pterosaurs bearing crests have them on both the skull and mandible, such as *Tapejara* and *Tupandactylus*, or the skull only, such as *Pteranodon* or *Nyctosaurus*. *Ikrandraco* is an exception, it only has a mandibular crest. Some hypothesize that a ventral crest on the mandible may have aided in the acquisition of prey by facilitating temporary skimming, where the mandible would be partially immersed and the crest would break the water, while pterosaurs lacking a crest could still engage in skim-feeding due to similarities with the skimming bird *Rynchops* [66]. However, many others have suggested that skim-feeding would not be possible in pterosaurs due to biomechanical constraints [8,67,68]. It is more commonly believed that the crests (especially cranial crests) were socio-sexual display structures, either being sexually dimorphic structures (e.g. [69,70] or mutually sexually selective features [71]. Cranial crests have been shown to develop in a positively allometric fashion, being larger in individuals presumably at, or reaching, sexual maturity. This has been reported in the tapejarid *Caiuajara* [72], the pteranodontian *Pteranodon* [73] and anhanguerids [34]. As cranial crests are linked to socio-sexual selection, it is reasonable to believe that, in the absence of clear biomechanical or functional evidence, mandibular crest development may also be driven by socio-sexual selection [71]. While the effect of cranial crests on aerodynamics has been studied previously [25], this has not been explored in mandibular crests. A mandibular crest would have affected both drag and yaw, but there is currently no evidence that aerodynamics was responsible for morphological innovations in mandibular crests. Therefore, we present evidence that socio-sexual selection was responsible for generating considerable geometric disparity in pterosaur mandibles, perhaps more so than dietary and functional factors.

### 4.2. Functional signal

There is some functional signal in variation along PC2 in both mandibular morphospaces, with regards to the position of the quadrate–articular joint relative to the occlusal plane (figures 3 and 4). In most pterosaurs, the mandible is a straight beam, and the jaw joint is positioned level with the tooth row. However, there are some basal, non-pterodactyloid, pterosaurs with high negative PC2 scores, representing mandibles where the joint is positioned below the tooth row (e.g. *Jianchangnathus*, *Raeticodactylus* and *Scaphognathus*). Having jaw joints positioned below the tooth row affect the occlusion between the upper and lower teeth [42], and the architecture of the adductor musculature needed to produce rapid jaw closure [11]. Two other functionally distinct morphotypes are positioned at the opposite end of PC2, in regions of high positive PC2 scores. The distinctive ctenochasmatid *Pterodaustro* had a uniquely curved and scoop-like jaw housing densely packed filament-like teeth, allowing efficient filter-feeding [22]. Also positioned at the positive extreme of PC2 is the dsungaripterid *Dsungaripterus*, which had a slender and distinctly upturned edentulous mandible tip, potentially used for prey acquisition before the prey was moved to more posteriorly positioned crushing teeth [74].

### 4.3. Size trends

There is no clear trend of size distributions in pterosaur mandibular morphospace. Statistical tests confirm that there is no overriding allometric pattern. Larger pterosaurs are widely distributed along both major morphospace axes, suggesting that aerodynamic and other functional restrictions did not confine all larger pterosaurs to a small area of overall mandibular morphospace, when compared with small- and medium-sized forms. The evolution of exceptionally large body size in some pterosaurs has been attributed to competition from Cretaceous birds [31], although other evidence suggests this is not the case [75,76]. This body-size expansion was not associated with any major innovations in jaw shape evolution or major shifts in morphospace.

### 4.4. Morphometric protocols: the use of landmarks and outlines

Our results show that outline and landmark-based morphometric techniques reveal congruent patterns. Similar morphological trends are recovered on the major axes of variation in multivariate morphospace, and in the preordination data. Other researchers have reached similar conclusions in other groups, while some have emphasized that the complexity of the structure may be pivotal [77–80]. We suggest that both methods may be interchangeable in simple biological structures which can be measured in two dimensions. The use of outline-based techniques may be a suitable alternative to a combined landmark and SLM approach when homologous landmarks cannot be accurately identified, or they are very sparse.

### 4.5. Future directions

Here we identify major shape innovations in pterosaur lower jaw evolution and explore potential drivers and constraints. Future studies may wish to investigate functional trends, to understand ecological partitioning within a biomechanical framework and provide insights into the relationship between ecology, geometry and biomechanical performance (e.g. [81,82]). This would be improved by additional studies of pterosaur jaw musculature, facilitating the use of biomechanical characters and potentially finite-element analysis. Dental microwear analysis also represents a potentially rich source of ecomorphologically relevant data [83–85].

## Supplementary Material

Electronic Supplementary Information

## Supplementary Material

Procrustes aligned landmarks coordinates

## Supplementary Material

Fourier coefficients

## Data accessibility

Supplementary information has been provided as the electronic supplementary material.
